# A century of structures: historical fidelity and computational fitness in the Inorganic Crystal Structure Database (ICSD)

**DOI:** 10.1107/S2052520626007079

**Published:** 2026-08-10

**Authors:** Peter Gross, Nik Reeves-McLaren

**Affiliations:** a School of Chemical, Materials and Biological Engineering University of Sheffield Mappin Street Sheffield S1 3JD United Kingdom; Moscow State University, Russian Federation

**Keywords:** bond valence sum, global instability index, site occupancy, cation disorder, structural validation

## Abstract

The earliest entries in the Inorganic Crystal Structure Database (ICSD) are both primary historical documents and active computational inputs. They record how the Braggs, Goldschmidt, Vegard, and others established the structural foundations of inorganic chemistry; they are also served, without distinction, to automated pipelines, machine-learning training sets, and high-throughput workflows. We assess 464 ICSD structures from 1913 to 1929 using two independent tools: *Eir v1.4.2* (bond valence sums, global instability index) and *MaplePy* (Madelung part of lattice energy). The pre-1925 median global instability index is 0.077 valence unit (v.u.), below that of modern rock salt reference data (mean 0.192 v.u.); the Braggs’ 1913 NaCl gives 0.005 v.u., Bragg’s 1922 corundum 0.001 v.u. Early crystallographers were doing simple structures and getting them right. The problem is not quality but metadata. A small number of entries carry genuine topological errors (BeO in NaCl type, SnTe in zinc blende) whose corrective information already exists in database Comments fields but is not machine-readable, not reflected in quality classifications, and relatively invisible to downstream users. We propose six automated fitness-for-purpose flags that surface these cases without removing or modifying any historical record.

## Abstract 2

Of 464 Inorganic Crystal Structure Database (ICSD) structures from 1913 to 1929, the vast majority are structurally sound, but a small number carry genuine topological errors whose corrective information exists in the database yet is invisible to automated queries. Six machine-readable fitness-for-purpose flags, which can be populated using existing tools without modifying any historical record, would bring ICSD into line with the quality metadata already provided by the CSD and the ICDD Powder Diffraction File.

## 1. Introduction

In June 1913, W. L. Bragg published the structure of NaCl: sodium and chlorine ions arranged on interpenetrating face-centred cubic lattices, with a bond length of 2.82 Å, determined from X-ray diffraction data alone (Bragg & Bragg, 1913 ▸). The topology has not been revised since. In that single determination, Bragg showed that the internal geometry of matter was directly measurable for the first time in the history of science. The fifteen years that followed produced a sequence of structure determinations that constitutes the primary structural reference set of inorganic chemistry: diamond, ZnS, fluorite (CaF_2_), MgO, spinel, corundum, rutile, pyrite, calcite. The people who determined these structures (the Braggs, Wyckoff, Goldschmidt, Vegard and others) were not refining an established methodology; the analytical framework of structural crystallography was developed through the determinations themselves.

Those structures are now Inorganic Crystal Structure Database (ICSD) entries. The NaCl determination that opened the field is ICSD collection code 53815. The spinel determination by Bragg (1915 ▸) is in the database. The pyrite determination by Bragg & Bragg (1913 ▸) is there. The diamond structure, resolved using a few dozen X-ray intensities and geometric intuition, is there alongside structures solved from synchrotron data collected at 100 K with *R* factors of 0.008. All of these entries are served identically to any query.

This is not a criticism of ICSD. Retrospective digitization of the crystallographic literature was an extensive undertaking, and the database that resulted is an irreplaceable scientific resource. The point is that the early entries are a particular kind of resource that current metadata systems were not designed to represent. They are primary historical documents: the first experimental records of structures that are now taught in every undergraduate inorganic chemistry course, widely cited, and used as benchmarks by every major materials database. A historian consulting Newton’s *Principia* in a critical edition does not treat it as methodologically equivalent to a twenty-first-century orbital mechanics calculation. The critical editor notes the date, the instruments available, the state of prior knowledge, and the subsequent corrections. ICSD currently has no mechanism for doing any of this, and the result is that the standard model of computational database use (a structure queried from ICSD is a structure, full stop) applies identically for entries from 1920 and 2020.

For most early entries, this matters little: the structures are correct, and modern analyses confirm that early crystallographers, working on the simplest available compounds, obtained correct results. There are, however, some localized problems that are consequential: a small fraction of early entries contain topological assignments that the original authors themselves identified as uncertain, or that subsequent work definitively corrected, and that information is either absent from the ICSD record or present only in a Comments field.

A case that makes this concrete is ICSD 26957: BeO, determined by Gerlach (1922 ▸). The entry is tagged room temperature, atmospheric pressure, standard quality, space group *Fm*3*m* (NaCl type). Gerlach’s original paper tells a different story. The reflections observed during the experiments were very broad, the sample was contaminated with an Al_2_O_3_ side phase, and quantification of the line intensity by photometric analysis had to be forgone, as the necessary equipment was only borrowed from Max von Laue and had to be handed back before the conclusion of the experiments. The assignment to the NaCl structure type was tentatively made on the basis of relative reflection intensities, and the high uncertainty of this approach as well as the observation of an additional reflection that suggested the ZnS type instead were explicitly acknowledged. The ICSD comments field records ‘Given sp. gr. was F23’, a reclassification from Gerlach’s original assignment. The ambient structure of BeO, established conclusively by the late 1920s, is wurtzite (*P*6_3_*mc*); three independent ICSD entries from 1925 to 1927 assign it correctly. Rock salt BeO is predicted to become the stable polymorph only above *ca* 90–105 GPa (Boettger & Willis, 1996 ▸; Cai *et al.*, 2006 ▸); the transition has not been observed experimentally.

What is instructive here is the epistemic record. Gerlach was honest in print: he acknowledged the ambiguity, gave his reasoning, and noted that ZnS-type could not be ruled out. The database inadvertently replaces his documented uncertainty with confidence he did not claim. This has consequences; the ICSD entry for a 1922 determination is also a primary historical source: a record of what was known, what was uncertain, and how conclusions were reached at a specific moment in the development of the field. Presented without that context, it becomes a datum rather than a document. The distinction matters for historians of science reading it as evidence of early crystallographic practice, for researchers treating it as a benchmark, for students encountering it as a model of how scientific conclusions are reported, and for any automated pipeline that returns it as an ambient-condition BeO structure. In each case, the weakness or absence of fitness-for-purpose metadata produces the same effect: Gerlach’s careful qualification is invisible, and the structure he doubted is served with the same authority as one he did not.

ICSD 26957 has since been reported to FIZ Karlsruhe, who confirmed same-day that the entry will be deleted. That response is welcome and reflects their commitment to data quality. It also illustrates the limitation of the current corrective mechanism: a century-old error in a binary oxide was identified by a single reader, reported individually, and resolved by manual intervention. Similar cases identified in this study remain in the database. A fitness-for-purpose metadata layer would surface all of them simultaneously, without requiring that each be found by a single reader.

## 2. Methods

### 2.1. Dataset compilation

ICSD was queried for all inorganic crystal structures with publication year 1929 or earlier. CIF files were exported in two batches (222 pre-1925 entries and 475 from 1925 to 1929, reflecting ICSD’s export limit), giving 697 structures in total. After filtering for measurement conditions classified as ambient (temperature below 350 K and pressure below 0.0002 GPa) or unknown (no temperature or pressure recorded in the CIF), 121 pre-1925 and 343 from 1925 to 1929 were retained, forming a single dataset of 464 structures. No structures were excluded after this point; all flags and anomalies reported below emerged from the tools.

### 2.2. Data analysis

Bond valence sums (BVS) were calculated using *Eir* (v1.4.2; Reeves-McLaren, 2026 ▸), which implements the formalism of Brown & Altermatt (1985 ▸) as extended by Brown (2009 ▸), using bond valence parameters from Gagné & Hawthorne (2015 ▸, 2016 ▸). The global instability index (GII) is defined following Salinas-Sanchez *et al.* (1992 ▸) as the root-mean-square deviation of all site BVS values from their formal valences, normalized per atom. *Eir* assigns eight error flags; of these, flag_type7 (charge balance irresolvable from CIF-assigned oxidation states, in practice identifying coordination complexes, polyoxyanion compounds, and structures where unusual element-oxidation state combinations lack BVS parameters) and flag_type8 (coordination environment outside the BVS parameterization domain) are the most relevant to historical data.

The MAdelung Part of Lattice Energy (MAPLE), introduced by Hoppe (1966 ▸, 1970 ▸, 1995 ▸) as a transferable thermodynamic fingerprint for ionic crystal structures, provides a diagnostic orthogonal to BVS. Where GII measures local bonding strain by comparing calculated and expected bond valences at each site, MAPLE encodes the global electrostatic energy of the crystal through the Ewald summation, normalized to a reference bond distance. For isostructural compounds, MAPLE / *d*_ref_ is approximately constant: MgO and CaO, both *Fm*3*m*, give MAPLE / *d*_ref_ = 2321.2 kcal mol^−1^ Å^−1^. Departures from isostructural trends flag structures where the reported topology or bond geometry is internally inconsistent. MAPLE is blind to topological errors that preserve the local charge balance: NaCl-type BeO [ICSD 26957 (Gerlach, 1922 ▸)] sits on the alkaline earth oxide trend because the Madelung factor is fixed for any rock salt topology regardless of which ions occupy it. Used in conjunction with GII, this complementarity defines the analytical scope of the combined approach.

*MaplePy* is an open-source Python tool (Gross & Reeves-McLaren, 2026 ▸, submitted) that automates MAPLE calculation from CIF files at batch scale, using pymatgen’s EwaldSummation class (Ong *et al.*, 2013 ▸). Oxidation states are assigned through a five-level priority chain designed to prevent silent failures; the implementation, validation against the analytical NaCl Madelung constant and Hoppe’s tabulated TiO_2_ polymorph values, and the additivity residual methodology are described in detail by Gross & Reeves-McLaren (2026 ▸, submitted). The MAPLE value (kcal mol^−1^) is obtained from the Ewald energy via MAPLE = *E*_Ewald_ × 23.0609; the reference bond distance *d*_ref_ is the shortest cation–anion distance, following Hoppe’s original definition. The reduced MAPLE M* = MAPLE / *d*_ref_ ( kcal mol^−1^ Å^−1^) is reported alongside total MAPLE for comparative analysis across structure types.

Structures for which all assigned oxidation states are zero are classified as non-ionic and returned with MAPLE = 0. This is correct behaviour: elemental metals, alloys, and noble gases are outside the domain of applicability of the ionic point-charge model. Of 161 unique pre-1925 compounds, 48 (29.8%) fall into this category; of 347 unique 1925–1929 compounds, 40 (11.5%) do. The declining fraction reflects the shift from simple metals and alloys in the earliest determinations towards the ionic oxides, halides, and sulfates that dominate the later period.

### 2.3. Data classification

Based on the combined GII and MAPLE results, each structure was assigned to one of three categories. Category 1 (genuinely problematic) covers structures where GII is elevated for reasons traceable to real topological or positional errors: wrong structure type assignment, incorrect lattice[unit-cell parameters, or positional errors that compress or extend bonds systematically. MAPLE provides a complementary diagnostic in this category where the topological error changes the Madelung sum – as in SnTe, where the zinc blende Madelung factor (1.6381) directly identifies the wrong structure type – but is blind where the Madelung sum is topology-independent, as in rock salt BeO. Category 2 (topologically correct, tool-inappropriate) covers structures where GII is elevated for reasons attributable to known limitations of BVS parameterization – directional bonding in mercury halides, soft-lattice tellurides, layered van der Waals compounds – rather than to structural error. Category 3 (outside both tools’ domain) covers structures for which the ionic point-charge model underpinning both BVS and MAPLE is inapplicable by definition: mixed-valence spinels, coordination complexes, molecular anion compounds (carbonates, sulfates, silicates), cyanide frameworks, and elemental or alloy structures. In the corpus, 136 structures (29.3%) carry one or both parameter coverage flags, marking the outer boundary of the combined analytical scope.

## 3. Results

### 3.1. GII distribution and comparison with modern datasets

A perhaps counterintuitive result is that early structures have lower GII than the relatively more modern ones. Pre-1925 structures (*n* = 121) show median GII of 0.077 v.u. (mean 0.160 v.u., standard deviation 0.202 v.u.), compared with a mean of 0.192 v.u. for ~700 ambient rock salt structures (Reeves-McLaren, 2026 ▸). The 1925–1929 period (*n* = 343) shows median 0.168 v.u. (mean 0.265 v.u., standard deviation 0.395 v.u.).

The explanation is compositional selection. Early crystallographers chose the simplest available structures: 43.8% of pre-1925 entries are *Fm*3*m* (NaCl-type, with ideally one free parameter), drawn from only 21 unique space groups. The rock salt dataset that underpins our GII reference value spans ∼700 structures across diverse compositions (transition metal oxides, alkali halides, alkaline earth chalcogenides), all in *Fm*3*m* with a single free parameter. Even within that constrained topology, the modern dataset shows a higher mean GII than the pre-1925 corpus, because it includes compositions with bonding environments (*e.g.* Jahn–Teller active cations, heavy chalcogenides) for which the BVS parameterization is under greater strain.

The 1925–1929 period shows this transition directly. The *Fm*3*m* fraction falls from 43.8% to 21.3% as the community moved to more complex targets: rutile (*P*4_2_/*mnm*, 28 structures), bixbyite (*I*2_1_3, 24), corundum (*R*3*c*, 11), and pyrite (*Pa*3, 11). The 55 unique space groups in the 1925–1929 data, compared with 21 in the earlier period, reflect the expansion of the structural repertoire, not a deterioration in experimental practice. The fraction of structures with GII above 0.20 v.u. rises from 21.5% (26/121) to 42.6% (146/343), but the molecular complexes, sulfates with missing S^6+^ BVS parameters, and layered halides newly represented in the later period contribute elevated GII values for reasons unrelated to experimental quality.

Within the 1925–1929 period, no systematic improvement in GII is detectable by year (1925: *n* = 63, mean GII 0.260 v.u.; 1926: *n* = 85, 0.208; 1927: *n* = 73, 0.267; 1928: *n* = 82, 0.333; 1929: *n* = 40, 0.252). The variation tracks compositional shifts: the *Fm*3*m* fraction drops to 3.7% in 1928, the year with the highest mean GII, while the number of unique space groups per year remains stable at 20–22. *R* factors were not a feature of structural crystallography until the mid-twentieth century; all pre-1930 entries therefore lack the primary quality indicator used by every modern database query. Summary statistics for both periods are given in Table 1 ▸.

The statistical picture is clear: these structures are, as a class, sound. The early crystallographers were studying simple structures and, generally, getting them right. What the statistics cannot convey is which individual entries are exceptions, and it is those exceptions that matter for any user, human or automated, who encounters them without warning.

### 3.2. Three-category classification: exemplars

The statistics above describe a population; they do not describe individual entries. A median GII of 0.077 v.u. tells a user that most pre-1925 structures are sound, but it does not identify the ones that are not. Here, we separate the corpus into structures where the tools detect a genuine problem (Category 1), structures where the tools report elevated values for reasons attributable to their own domain limitations rather than structural error (Category 2), and structures where neither tool is applicable (Category 3). The exemplars below illustrate what each category looks like in practice, and what the database currently does with them.

Category 1: genuinely problematic structures. The defining feature of Category 1 is that the database presents a structure that any user will interpret as a correct ambient-condition determination, and it is not. Selected Category 1 structures are summarized in Table 2 ▸.

The clearest case is SnTe (ICSD 53956), assigned by Goldschmidt (1927 ▸) to *F*43*m* [zinc blende, coordination number (CN) = 4 for Sn]. The ambient-condition ground state of SnTe is NaCl-type (*Fm*4*m*, CN = 6); a correct determination exists in the database as ICSD 52489 (Rogacheva *et al.*, 1986 ▸). GII = 1.14 v.u. (BVS = 4.29, expected 2.0: discrepancy 114%) identifies the problem from the BVS side; the MAPLE Madelung factor of 1.638, equal to the analytical zinc blende value (1.6381) rather than the NaCl value (1.7476), identifies it independently from the electrostatic side. The Comments field of ICSD 53956 records ‘See ICSD52489 in Fm3-m (NaCl-type)’: the database contains the correction. However, the cross-reference is not bidirectional (ICSD 52489 carries no reciprocal warning), quality metadata for both entries are Standard, and both are commented as being served to the Materials Project and the Open Quantum Materials Database (OQMD). An automated query for ambient SnTe may receive either structure, with no machine-readable indication that one is topologically wrong.

A second Category 1 pattern is inter-entry inconsistency. NbO_2_ appears twice in the 1925–1929 set, both in *P*4_2_/*mnm* (rutile type): ICSD 56018 gives GII = 0.066 v.u. (Nb BVS 4.27, discrepancy +6.6%), ICSD 645141 gives GII = 0.608 v.u. (Nb BVS 1.57, discrepancy −60.8%; Goldschmidt *et al.*, 1926 ▸). A GII spread of 0.54 v.u. for the same compound in the same space group is not a difference in structural chemistry: it is a difference in the reliability of the reported cell parameters or atomic positions. Any query returning either entry without flagging the other serves a conflict it does not acknowledge.

PbI_2_ (layered, *P*3*m*1) shows an analogous spread. ICSD 30347 gives GII = 2.20 v.u. with CN = 10 for Pb; ICSD 52370 (Ferrari & Giorgio, 1929 ▸) gives GII = 0.25 v.u. with the correct octahedral CN = 6. The CN = 10 entry aggregates interlayer Pb–I contacts into the coordination shell, most plausibly from unit-cell parameters that compress the *c*/*a* ratio relative to the correct highly anisotropic value. PbI_2_ is isostructural with CdI_2_; the correct coordination is not in dispute.

V_2_O_3_ (ICSD 33641, *R*3*c*, Goldschmidt *et al.*, 1928 ▸) presents a positional error in the corundum structure: the deposited oxygen fractional coordinates (−0.3, 0.8, 0.25), rounded to one decimal place without estimated standard deviations, compress all six V–O distances to 1.47 Å, roughly 25% shorter than the accepted values of 1.96–2.06 Å. GII = 0.841 v.u. The topology is correct; the coordinates are not.

The BeO case discussed in the introduction completes the Category 1 picture. Three BeO structures determined between 1925 and 1927 [ICSD 31072 (Aminoff, 1925 ▸), 31825 (Zachariasen, 1925 ▸), 56144 (Haase, 1927 ▸)] are present in the dataset, all assigned to wurtzite (*P*6_3_*mc*, CN = 4 tetrahedral). Their GII values are 0.025, 0.054, and 0.032 v.u.: among the lowest in the corpus. Gerlach’s (1922 ▸) report was corrected independently by at least three groups within five years, but the database currently presents four BeO entries with equal metadata weight. The self-correction that the scientific record documents, the database does not.

Category 2: topologically correct, outside BVS domain. Mercury(I) chloride (ICSD 157979, Hg_2_Cl_2_, *I*4/*mmm*; Havighurst, 1925 ▸) gives GII = 0.698 v.u. with no parameter coverage flag. Mercury halides have highly directional Hg–*X* bonding, and the Hg_2_^2+^ dimer additionally has a metal–metal bond; neither is captured by a BVS parameterization that treats bond valence as a monotonic function of bond length, The GII is elevated for real physical reasons, but those reasons are properties of the bonding, not errors in the structure. A fitness-for-purpose flag would distinguish this case from SnTe: both have high GII, but only one is wrong.

Category 3: outside both tools. Tris(hexammine)cobalt(III) iodide [ICSD 36309, GII = 0.977 v.u.; Stoll (1926 ▸)], alum KAl(SO_4_)_2_·12H_2_O [ICSD 56112, GII = 3.19 v.u.; Cork (1927 ▸)], and CsIO_3_ [ICSD 33665, GII = 4.51 v.u.; Goldschmidt *et al.* (1928 ▸)] all carry one or both of *Eir*’s parameter coverage flags. These are coordination complexes, molecular anion compounds, and structures where BVS parameters are absent from the Gagné–Hawthorne tables. The GII values are artefactual: CsIO_3_ holds the dataset maximum at 4.51 v.u. because I^5+^ parameters do not exist, not because the structure is strained. In the corpus, 136 structures (29.3%) are flagged for incomplete BVS coverage, marking the outer boundary of the combined analytical scope. For these entries, the appropriate metadata response is not a quality judgement but a domain declaration: the tools do not apply, and the GII should not be interpreted.

The cases above are exceptions. The corpus as a whole tells a different story.

### 3.3. The founding structures

The corpus contains a number of the structures on which inorganic crystallography was built. ICSD 53815 is the Braggs’ 1913 NaCl determination: the first crystal structure solved by X-ray diffraction, published in *Proceedings of the Royal Society*. Its GII is 0.005 v.u. Bragg’s 1922 corundum (ICSD 56085, *R*3*c*) gives GII = 0.001 v.u., the lowest in the pre-1925 corpus. Bragg’s 1915 spinel [ICSD 56116, *Fd*3*m*; Bragg (1915 ▸)] gives 0.104 v.u. Vegard’s 1916 rutile [ICSD 53997, *P*4_2_/*mnm*; Vegard (1916 ▸)] gives 0.085 v.u. Wyckoff’s (1920 ▸) calcite (ICSD 37241, *R*3*c*) gives 0.080 v.u. Gerlach’s 1922 fluorite and sphalerite (ICSD 53978 and 53943) give 0.032 and 0.021 v.u., respectively. Later entries from Goldschmidt *et al.* (1926 ▸) (MgO, 0.012 v.u.) and Pauling & Hendricks (1925 ▸) (corundum, 0.004 v.u.) continue the pattern. All fall well below the 0.20 v.u. threshold.

A distinction between accuracy and precision is useful here. The early determinations are accurate: the topologies are correct, the bond lengths have not been revised, and the GII values confirm that the reported geometries are internally consistent. They are not precise by modern standards: coordinates are reported to one or two decimal places without estimated standard deviations, and the work predated by decades the Rietveld method and the refinement statistics that would come much later. The modern database quality infrastructure is built almost entirely on precision metrics – *R* factors, e.s.d.’s, residual electron density maps – none of which these entries possess. The absence of precision metadata is the reason they fall outside the quality system; it is not evidence that they are wrong. The V_2_O_3_ case (Section 3.2) shows that imprecision can, in extreme cases, produce inaccuracy – but the GII distribution demonstrates that this is the exception rather than the rule.

These are not structures that happen to be old. They are the structures through which the field established its methods: the experimental reference points against which subsequent determinations were calibrated, the examples on which the theory of space group symmetry was tested, and in several cases the type structures from which entire classification systems derive. Bragg’s NaCl is the archetype for every rock salt compound in the database; his spinel is the archetype for all the isostructural entries that follow it. The fact that his 1913 bond length of 2.82 Å for NaCl has not been revised in over a century is not a curiosity: it is evidence that the measurement was correct. There is enduring scientific merit, as well as historical importance in the work.

What the database cannot convey is the context in which those measurements were made. A GII of 0.005 v.u. for NaCl in 2026 is unremarkable; for a structure solved in 1913 from photographic film, without the benefit of Fourier methods, least-squares refinement, or a prior example of any solved crystal structure, is truly special. Both carry identical ICSD metadata. The fitness-for-purpose gap identified earlier is one consequence of that equivalence; the loss of historical and pedagogical context is another.

## 4. Discussion

### 4.1. The metadata gap is structural, not incidental

The ICSD quality metadata system was designed for modern structures. The 2021 database documentation states that ‘only data that have passed thorough quality checks are included’. The quality levels observable in practice (High and Standard) are assigned on the basis of the *R* factor and internal consistency checks calibrated against post-1950 data. The quality classification itself is not exposed as a searchable or filterable field in any ICSD query interface: standard, advanced, or expert. Users can filter by *R* factor or search the Comments field as free text, but cannot construct a query that returns only entries of a specified quality level. For pre-1930 structures, which carry no *R* factor and whose Comments fields are unstandardized, neither workaround is available. The result is that pre-1930 entries do not fail the quality system: they fall outside it entirely, and emerge in query results as implicitly equivalent to modern determinations.

ICSD 26957 (BeO) and ICSD 53956 (SnTe) illustrate complementary failure modes. In both cases, the database contains the corrective information: for BeO, the Comments field records ‘Given sp. gr. was F23’; for SnTe, it records ‘See ICSD52489 in Fm3-m (NaCl-type).’ In neither case does the correction reach an automated query. Both entries carry Standard quality metadata. Both are linked to the Materials Project and OQMD. The SnTe cross-reference is not even bidirectional: ICSD 52489, the correct NaCl-type determination, carries no reciprocal warning about ICSD 53956.

The downstream implications extend beyond the database itself. The Materials Project and OQMD both ingest ICSD data and apply automated geometry optimization and property calculation. An incorrectly assigned BeO topology (NaCl instead of wurtzite) will be geometry-optimized by DFT, which may or may not converge to the correct ground state depending on the exchange-correlation functional and the magnitude of the initial positional displacement. If it does not converge, a wrong energy enters a phase diagram. If it does converge, the provenance chain attributes the corrected structure to a 1922 determination with no annotation of the correction. GNoME training sets that draw from the Materials Project inherit whatever errors were not caught by geometry optimization.

This is not an argument against using early crystal structures. It is an argument that the mode of use matters, and that the metadata should encode it.

### 4.2. A minimum fitness-for-purpose flag set

The analysis above points to six machine-readable flags that could be added to ICSD entries without requiring structural re-determination and without removing any existing data.

Boolean *R* factor availability flag. The absence of an *R* factor is directly interpretable: it means the entry predates the refinement era (likely pre-1940) or the original paper did not report one. This flag requires no calculation, only inspection of the existing *R*-factor field. GII threshold flag. A GII threshold of 0.20 v.u. is well established in the BVS literature as the boundary above which manual review is recommended (Brown, 2009 ▸). In this corpus, 21.5% of pre-1925 structures and 42.6% of 1925–1929 structures exceed it. Flagging them does not assert that they are wrong; it asserts that they warrant inspection. BVS parameter coverage, corresponding to *Eir*’s BVS parameter coverage flag: at least one element–oxidation state combination in the structure lacks parameterization. This flag is informative independently of the GII value: a structure with all BVS parameters and GII = 0.35 v.u. is a different diagnostic category from one missing half its parameters. MAPLE additivity residual. Where MAPLE is calculable, a residual above ±5% relative to the expected sum of binary reference values indicates internal inconsistency in the bond distances. The threshold is established empirically from Hoppe’s original work on MAPLE validity. Structure type ambiguity annotation. The BeO and SnTe cases demonstrate that the Comments field already contains the information a structured flag would encode: a reclassified space group for BeO, a cross-reference to the correct topology for SnTe. A structured flag populated by text parsing of existing Comments fields would surface both automatically, without requiring new analysis. An interlayer coordination warning for layered van der Waals compounds, applicable where the CN assigned by the structure file is inconsistent with the topological bonding (as in PbI_2_ ICSD 30347, where CN = 10 rather than the correct octahedral CN = 6).

The applicability of the proposed flags across the full ICSD can be estimated without running the tools on all ∼260000 entries. Flag 1 (*R* factor availability) requires only inspection of existing metadata fields and is immediately applicable at database scale. Flag 3 (BVS parameter coverage) is similarly metadata-derived: the Gagné–Hawthorne parameter tables define which element–oxidation state pairs are covered, and any entry containing a pair outside those tables would be flagged. In the present corpus, 29.3% of entries (136/464) carry this flag; the fraction for the full database will depend on the distribution of compound types but is unlikely to be lower, given that the ICSD contains substantial numbers of coordination complexes, organometallics, and molecular-anion compounds for which BVS parameters are incomplete. Flag 2 (GII threshold) requires a BVS calculation for each entry but is computationally inexpensive: *Eir* processes a single CIF file in under one second on commodity hardware, and a full ICSD run is feasible in hours. Flags 4 (MAPLE additivity residual) and 5 (structure type ambiguity) are similarly scalable; flag 6 (interlayer coordination) applies only to the subset of layered compounds and could be implemented as a conditional check triggered by space group and known structure type. The tools are designed for database-scale deployment; the present study demonstrates them on a historically significant subset, but no technical barrier prevents their application to the full ICSD.

None of these flags requires a judgement about whether a structure is correct. Each records the output of a defined test: a field is present or absent, a value is above or below a threshold, a parameter set is complete or incomplete. While the flags themselves are automatable, reproducible, and independent of expert interpretation of the primary crystallographic data, the authors acknowledge that identification of underlying reasons for the occurrence of individual flags as well as decisions about downstream data handling based on these interpretations certainly do benefit from a deeper understanding of crystallography and structural chemistry, as will be illustrated below. It is nevertheless our firm belief that a solid-state scientist’s limited time is best spent understanding these interesting cases, while leaving their mere identification to automated tools like *Eir* and *MaplePy*.

The intended function of the flags in automated pipelines is triage, not potentially careless or thoughtless exclusion. Flag 1 (no *R* factor) and flag 3 (incomplete BVS coverage) are pre-calculation filters: a pipeline that requires validated bonding geometry can exclude entries lacking either, reducing the ingested dataset to structures within the diagnostic domain before any property calculation begins. Flag 2 (GII above threshold) is a post-calculation diagnostic: it identifies entries whose bonding geometry is anomalous without prescribing a response, and an appropriate action will depend on the application. A machine-learning training set might exclude entries with GII above 0.50 v.u. while retaining those between 0.20 and 0.50 v.u. with reduced weighting; a phase diagram calculation might prioritize flagged entries for manual inspection before including their energies. Flag 4 (MAPLE additivity residual) serves a similar post-calculation function. Flag 5 (structure type ambiguity) and flag 6 (interlayer coordination) are warnings that a specific known problem has been detected in the entry metadata and that the user should consult the Comments field or companion entries before proceeding. The flags do not impose a single workflow; they provide the information that a workflow designer needs to make an informed decision about which entries to trust for a given purpose.

### 4.3. Comparison with CSD and ICDD practice

The Cambridge Structural Database handles historical data differently from ICSD. CSD entries carry explicit reliability annotations, and the CSD Python API exposes granular quality indicators including coordinate precision, disorder classification, and 3D coordinate availability flags. The CSD also maintains a systematic programme of retrospective corrections. These indicators are machine-readable and queryable: a user building an automated pipeline can filter by data quality before any calculation begins.

The ICDD’s Powder Diffraction File takes a different but equally systematic approach. Every entry in the PDF is editorially reviewed and assigned a quality mark (Star, Indexed, or Blank in decreasing order of data quality), with additional categories for Calculated and Rietveld-derived patterns (Gates-Rector & Blanton, 2019 ▸). The PDF also maintains Primary, Alternate, and Deleted status flags for multiple entries of the same phase, so that a user querying quartz, for example, can filter directly to the primary Star-quality pattern from among the 77 entries available. The editorial process is ISO 9001:2015 certified and has been computer-assisted since 1965. Quality marks are searchable and filterable in the standard query workflow.

ICSD occupies a different position. Quality classifications exist (High and Standard have been observed in entry records), but the classification criteria are not defined in the publicly accessible documentation, the quality field is not exposed in any search interface, and the system is calibrated against post-1950 data. For pre-1930 entries, the classification is inapplicable by design: no *R* factor, no refinement statistics, no basis for the consistency checks on which the quality scheme depends. The CSD and the PDF both solved the problem of historical data quality by building machine-readable quality indicators into their query infrastructure. ICSD has not.

The proposal here is correspondingly modest. It does not require FIZ Karlsruhe to build an editorial review system equivalent to the PDF’s, nor to re-examine early entries individually. It requires the addition of six Boolean or numeric fields to the ICSD schema, populated by automated calculation for GII, BVS coverage, and MAPLE, and by text parsing of existing Comments fields for structure type ambiguity annotations. The calculation burden is equivalent to one *Eir* run across the pre-1930 subset. The tools to populate the flags already exist; the question is whether the database schema will accommodate them.

### 4.4. Limitations of the two-tool approach

Both *Eir* and *MaplePy* operate under assumptions that limit their diagnostic power for early structures. BVS treats bond valence as spherically symmetric and additive, making it structurally blind to lone-pair effects (Sn^2+^, Bi^3+^, Pb^2+^ in non-centrosymmetric environments), directional and metal–metal bonding (mercury halides), and interlayer interactions in van der Waals compounds. MAPLE assumes the ionic point-charge model, excluding all structures with covalent or metallic character. The 136 structures (29.3%) carrying parameter coverage flags in this corpus include cyanide frameworks (K_2_Zn(CN)_4_, K_2_Cd(CN)_4_), hexa­chloro­platinates (K_2_PtCl_6_), and thio­cyanate coordination compounds for which neither tool has anything meaningful to say.

A further limitation specific to the GII is the dependence of bond valence sums on the assigned CN. For well ordered structures with unambiguous first-coordination-shell geometries, the coordination environment is uniquely defined by the crystal structure and the BVS calculation is reproducible. For distorted structures, particularly those containing large cations (Ba^2+^, Pb^2+^, K^+^) or ions with stereoactive lone pairs, the boundary of the first coordination shell is not uniquely defined and depends on the distance cutoff used to assign coordinating anions (Gagné & Hawthorne, 2016 ▸). *Eir* uses pymatgen’s Voronoi nearest-neighbour algorithm to assign coordination environments, which avoids a fixed distance cutoff but introduces its own sensitivity to Voronoi cell decomposition in highly distorted geometries. The PbI_2_ case discussed in Section 3.2 illustrates the consequence: ICSD 30347 returns CN = 10 for Pb because interlayer I contacts fall within the assigned coordination shell, whereas the correct topological coordination is octahedral (CN = 6). The GII threshold flag (flag 2) is therefore best understood as a triage tool rather than a definitive diagnostic: it identifies structures warranting inspection, but the inspection itself may require expert judgement about the appropriate coordination environment. These are but a few examples illustrating our previous statement that the flags are ‘independent of expert interpretation’ with regards to the flag assignment (which is automated and reproducible for a given tool and parameterization), but not to the structural interpretation of a flagged entry (Section 4.2).

The appropriate response to tool inapplicability is a flag, not silence. If *Eir* flags a structure because I^5+^ BVS parameters are absent, the database should encode that the GII value is unreliable, not leave a user to discover it by inspection. The CsIO_3_ entry (ICSD 33665, GII = 4.51 v.u.) would propagate as a severely strained structure into any database query that did not first check the parameter coverage flag. The tools do their jobs correctly; it is the absence of metadata to communicate their limitations that creates the problem.

## 5. Conclusion

The 464 ICSD entries from 1913 to 1929 are not, as a class, unreliable. The pre-1925 median GII of 0.077 v.u. is lower than the mean of 0.192 v.u. for ∼700 modern ambient-condition rock salt structures reported in a prior study (Reeves-McLaren, 2026 ▸), and the 1925–1929 period shows median GII of 0.168 v.u. Early crystallographers were doing simple structures and getting them right. The Braggs’ 1913 NaCl determination gives GII = 0.005 v.u.; Bragg’s 1922 corundum gives 0.001 v.u. These are not merely adequate for their time: they are correct by any modern standard.

The problem is not uniform quality degradation. It is that a small number of entries carry genuine topological errors (SnTe in zinc blende, BeO in NaCl type), in some cases with corrective information already present in the database Comments field but invisible to automated queries. It is also that the database, by treating all entries as equivalent data points, removes the historical and epistemic context that makes the early determinations interpretable as primary scientific documents. A 1913 structure solved from photographic film without Fourier methods carries identical metadata to a 2026 synchrotron refinement. The CSD and the ICDD’s Powder Diffraction File both provide machine-readable quality indicators for their holdings; ICSD does not.

The minimum corrective action is the addition of six machine-readable fitness-for-purpose flags: *R* factor availability, GII threshold, BVS parameter coverage, MAPLE additivity residual, structure type ambiguity, and interlayer coordination consistency. No existing entry need be removed or modified. The historical record is preserved exactly as deposited. The flags encode what the tools can determine: not whether a structure is correct, but whether it has passed or failed the diagnostics available in 2026. Further flags could address additional classes of structural error not captured by the present tools; overlooked centrosymmetry, the systematic misassignment of non-centrosymmetric space groups to structures that possess an inversion centre, is one well documented example (Marsh, 1995 ▸). Automated symmetry checking is already implemented in some refinement packages and could be applied retrospectively at database scale.

Fitness-for-purpose metadata would serve both functions: protecting automated pipelines from the small number of genuinely problematic entries, and preserving the epistemic context that makes the founding structures interpretable as primary scientific documents. A community standard for fitness-for-purpose metadata in historical database entries, analogous to the FAIR principles for research data but specific to crystallographic fitness-for-computational-use, would extend this proposal beyond the ICSD to all major structure databases. The data to support it, and the tools to populate it, already exist.

## Supplementary Material

The raw Eir analysis on which the paper is based, shared to allow reproduction of findings. DOI: 10.1107/S2052520626007079/yh5050sup1.xlsx

## Figures and Tables

**Table 1:** Summary statistics for pre-1925 (*n* = 121) and 1925–1929 (*n* = 343) ICSD structures GII, lacking BVS parameters, and *R* factor availability are from *Eir* v1.4.2; GII thresholds follow Brown (2009 ▸). MAPLE ionic fractions are from *MaplePy*; non-ionic compounds (elemental metals, alloys, noble gases) return MAPLE = 0 by definition and are classified rather than calculated.

Parameter	Pre-1925	1925–1929
*n* structures	121	343
Unique space groups	21	55
*Fm*3*m* fraction (%)	43.8	21.3
Median GII (v.u.)	0.077	0.168
Mean GII (v.u.)	0.160	0.265
GII > 0.20 v.u. (%)	21.5	42.6
GII > 0.50 v.u. (%)	9.9	11.7
BVS parameters absent (%)	21.5	18.7
*R* factor available (%)	0	0
MAPLE: ionic compounds (%)	70.2	88.5

**Table 2:** Selected Category 1 (genuinely problematic) structures from the 1925–1929 dataset, with GII, MAPLE Madelung factor (where calculable), and assigned/correct topology

ICSD	Formula	Space group (assigned)	Correct topology	GII (v.u.)	Madelung factor	Diagnostic
26957	BeO	*Fm* 3 *m*	*P*6_3_*mc* (wurtzite)	0.382	1.748 (NaCl)	Wrong topology; MAPLE = 1223 kcal mol^−1^, matches isostructural series
53956	SnTe	*F*43*m*	*Fm*3*m* (NaCl)	1.143	1.638 (ZnS)	Wrong topology; Madelung factor identifies zinc blende
645141	NbO_2_	*P*4_2_/*mnm*	Same	0.608	n/a	Positional errors; companion entry 56018 GII = 0.066 v.u.
30347	PbI_2_	*P*3*m*1	Same	2.195	n/a	CN = 10 versus correct 6; companion entry 52370 GII = 0.245 v.u.
33641	V_2_O_3_	*R* 3 *c*	Same	0.841	n/a	Positional error; O fractional coordinates deposited to one decimal place, giving V–O = 1.47 Å versus accepted 1.96–2.06 Å

## References

[bb1] Aminoff, G. (1925). *Z. Kristallogr.* **62**, 113–122.

[bb50] Boettger, J. C. & Wills, J. M. (1996). *Phys. Rev. B* **54**, 8965–8968. 10.1103/physrevb.54.8965 9984608

[bb2] Bragg, W. H. (1915). *London Edinb. Dubl. Philos. Mag. J. Sci.* **30**, 305–315.

[bb3] Bragg, W. H. & Bragg, W. L. (1913). *Proc. R. Soc. London Ser. A, Containing Pap. Math. Phys. Charact.* **88**, 428–438.

[bb4] Brown, I. D. (2009). *Chem. Rev.* **109**, 6858–6919. 10.1021/cr900053k PMC2791485 19728716

[bb5] Brown, I. D. & Altermatt, D. (1985). *Acta Cryst.* B**41**, 244–247.

[bb51] Cai, Y., Wu, S., Xu, R. & Yu, J. (2006). *Phys. Rev. B* **73**, 184104.

[bb6] Cork, J. M. (1927). *London Edinb. Dubl. Philos. Mag. J. Sci.* **4**, 688–698.

[bb7] Ferrari, A. & Giorgio, F. (1929). *Atti Accad. Naz. Lincei R. Cl. Sci. Fis. Mater. Nat.* **10**, 522–527.

[bb8] Gagné, O. C. & Hawthorne, F. C. (2015). *Acta Cryst.* B**71**, 562–578. 10.1107/S2052520615016297 PMC4591556 26428406

[bb9] Gagné, O. C. & Hawthorne, F. C. (2016). *Acta Cryst.* B**72**, 602–625. 10.1107/S2052520616008507 PMC4971548 27484381

[bb10] Gates-Rector, S. & Blanton, T. N. (2019). *Powder Diffr.* **34**, 352–360.

[bb11] Gerlach, W. (1922). *Z. Phys.* **9**, 184–192.

[bb12] Goldschmidt, V. M. (1927). *Skr. Nor. Vidensk.-Akad. [Kl.] 1: Mater.-Naturvidensk. Kl.* **8**, 1–156.

[bb13] Goldschmidt, V. M., Barth, T. F. W., Holmsen, D., Lunde, G. & Zachariasen, W. H. (1926). *Skr. Nor. Vidensk.-Akad. [Kl.] 1: Mater.-Naturvidensk. Kl.* **9**, 1–17.

[bb14] Goldschmidt, V. M., Barth, T. F. W. & Lunde, G. V. V. (1928). *Skr. Nor. Vidensk.-Akad. [Kl.] 1: Mater.-Naturvidensk. Kl.*, pp. 1–165.

[bb15] Gross, P. & Reeves-McLaren, N. (2026). *ChemRxiv*, https://chemrxiv.org/doc/84c976b9-5811-4d18-9cf8-37273b4a6f81.

[bb16] Haase, M. (1927). *Z. Kristallogr.* **65**, 510–587.

[bb17] Havighurst, R. J. (1925). *Am. J. Sci.* **s5-10**, 15–28.

[bb18] Hoppe, R. (1966). *Angew. Chem. Int. Ed. Engl.* **5**, 95–106.

[bb19] Hoppe, R. (1970). *Angew. Chem. Int. Ed. Engl.* **9**, 25–34.

[bb20] Hoppe, R. (1995). *Z. Naturforsch. A* **50**, 555–567.

[bb21] Marsh, R. E. (1995). *Acta Cryst.* B**51**, 897–907.

[bb22] Ong, S. P., Richards, W. D., Jain, A., Hautier, G., Kocher, M., Cholia, S., Gunter, D., Chevrier, V. L., Persson, K. A. & Ceder, G. (2013). *Comput. Mater. Sci.* **68**, 314–319.

[bb23] Pauling, L. & Hendricks, S. B. (1925). *J. Am. Chem. Soc.* **47**, 781–790.

[bb24] Reeves-McLaren, N. (2026). *Dalton Trans.* **55**, 2868–2882. 10.1039/d5dt03003j 41614364

[bb25] Rogacheva, E. I., Gorne, G. V., Laptev, S. A., Arinkin, A. V. & Vesiene, T. B. (1986). *Izv. Akad. Nauk SSSR Neorg. Mater.* **22**, 41–44.

[bb26] Salinas-Sanchez, A., Garcia-Muñoz, J. L., Rodriguez-Carvajal, J., Saez-Puche, R. & Martinez, J. L. (1992). *J. Solid State Chem.* **100**, 201–211.

[bb27] Stoll, P. (1926). Diss. ETH Zürich, pp. 1–46.

[bb28] Vegard, L. (1916). *London Edinb. Dubl. Philos. Mag. J. Sci.* **32**, 505–518.

[bb29] Wyckoff, R. W. G. (1920). *Am. J. Sci.* **s4-50**, 317–360.

[bb30] Zachariasen, W. H. (1925). *Nor. Geol. Tidsskr.* **8**, 189–200.

